# Effects of general anaesthesia with an inhalational anaesthetic agent on the expression of exosomes in rats

**DOI:** 10.7150/ijms.72565

**Published:** 2022-08-08

**Authors:** Liyun Piao, Og-Heui Na, Eun-Hye Seo, Seung-Wan Hong, Kyo-Min Sohn, Yubi Kwon, Seung-Hyun Lee, Seong-Hyop Kim

**Affiliations:** 1 Department of Infection and Immunology, Konkuk University School of Medicine, Seoul, Korea.; 2 Department of Medicine, Jeju National University Graduate School, Jeju, Korea; 3 BK21 plus, Department of Cellular and Molecular Medicine, Konkuk University School of Medicine, Seoul, Korea.; 4 Department of Anesthesiology and Pain medicine, Konkuk University Medical Center, Konkuk University School of Medicine, Seoul, Korea.; 5 Department of Microbiology, Konkuk University School of Medicine, Seoul, Korea.; 6 Department of Medicine, Institute of Biomedical Science and Technology, Konkuk University School of Medicine, Seoul, Korea.

**Keywords:** Exosome, General anaesthesia, Isoflurane, CD63, CD81

## Abstract

**Background:** We hypothesized that the expression of exosomes under general anaesthesia with an inhalational anaesthetic agent would be changed. The study was designed to confirm the effect of general anesthesia with an inhalational anaesthetic agent on the expression of exosomes in rats.

**Methods:** After intraperitoneal administration for the mixture of ketamine and xylazine, tracheal intubation was performed. Just before the connection to ventilator, Control group and Anaesthesia group, according to anaesthesia with isoflurane, were allocated. The expressions of exosomes were checked in bronchoalveolar lavage (BAL), the blood and the tissues from the lung and the brain. Cytokines in the blood were also assessed.

**Results:** The expressions of cluster of differentiation (CD)63 and CD81 as markers for the exosomes in the blood was increased after anaesthesia with isoflurane (CD63, 0.078 ± 0.057 % in Control group *vs.* 0.180 ± 0.036 % in Anaesthesia group, *p* = 0.02; CD81, 0.028 ± 0.034 % in Control group *vs.* 0.245 ± 0.054 % in Anaesthesia group, *p* < 0.01). However, the increased expression of them were not checked in BAL, and the tissues from the lung and the brain. The cytokines in the blood did not show any significant difference before and after anaesthesia with isoflurane.

**Conclusion:** General anaesthesia with an inhalational anaesthetic agent increased the expression of exosomes in the blood. However, the change was limited in the blood, not the alveoli and the brain.

## Introduction

The exact mechanism of inhalational anaesthetic agents is not completely known. The inspired inhalational anaesthetic agent is delivered into the alveoli and is passively diffused into the blood. The inhalational anaesthetic agent is distributed in the blood to the brain. The inhalational anaesthetic agent is thought to affect communication between the alveoli, the blood, and the brain 1.

Exosomes are extracellular vesicles that carry cell-specific proteins, lipids, and genetic cargos. By merging a recipient cell, cell-specific proteins, lipids, and genetic cargos in exosomes from one cell is transferred to the recipient cell. The transferred contents in exosomes affect protein production, regulating biological process 2. Numerous studies have confirmed that exosomes play key roles in the communication between cells 3-5. They have found that exosomes preserve homeostasis in normal condition and mediate immune responses. Barile reported that exosomes have shown cardioprotective effect 6,7. Exosomes in cardiac surgeries were associated with the clinical course 8-11. Habertheuer et al. reported that exosome was able to be non-invasively used for early detection of acute rejection in lung transplantation 12. The findings regarding exosomes were not limited in cardiothoracic diseases. Many researches, using exosomes, have been performed and shown the productive results in various diseases, including tumours, neurodegenerative diseases and so on, for diagnostic or therapeutic tool 13-15. Inhalational anaesthetic agent in general anaesthesia is delivered into the respiratory system and expected to get the impact on the respiratory system. Stassen et al. reported that the exposure of the respiratory stressor induced surface markers of exosomes, such as cluster differentiation (CD)63 and CD81 16. However, research on the effects of general anaesthesia with an inhalational anaesthetic agent on exosomes is rare.

We hypothesised that general anaesthesia with an inhalational anaesthetic agent would change the expression of exosomes in the alveoli, blood, and brain. The study was designed to confirm the effect of general anaesthesia with an inhalational anaesthetic agent on the expression of exosomes, using CD63 and CD81 as the classical surface markers for exosomes, by alveoli, the blood, and the brain of rats.

## Materials and Methods

After approval from Institutional Animal Care and Use Committee (IACUC), Konkuk University (approval number: KU19134), Seoul, Korea, all experiments were conducted at Konkuk University Laboratory Animal Research Centre, Seoul, Korea, following IACUC guidelines and National Institutes of Health Guidelines for Care and Use of Laboratory Animals. The datasets used and analyzed during the current study are available from the corresponding author (Seong-Hyop Kim, yshkim75@daum.net) on reasonable request.

### Animal preparation

Adult male 8-week-old Sprague-Dawley rats were purchased from Orient Bio (Seongnam, Korea). All rats were housed in cages with free access to water and food, and acclimated to the condition of the study for 7 days before the study. A mixture of ketamine (Yuhan, Seoul, Korea) 80 mg/kg and xylazine (Bayer Korea Ltd., Seoul, Korea) 10 mg/kg were intraperitoneally administered for anaesthesia induction. The rats were intubated with a 16 gauge catheter (BD, Franklin Lakes, NJ, USA) in the supine position. The catheter was intubated through the larynx into the bronchus. A ventral incision was performed in the neck and the trachea was exposed. A surgical tie was made around the trachea with 5-0 silk for the prevention of air leakage. The correct position for the catheter was confirmed with symmetrical chest expansion 17,18. Controlled mechanical ventilation was conducted, using a ventilator (Harvard Apparatus, Holliston, MA, USA): 1) fraction of inspired oxygen, 0.4 with flow rate, 0.4 L/min; 2) tidal volume, 6 ml/kg; 3) inspiration and expiration ratio, 1:1; 4) respiration rate, 50 breaths/min; and 5) positive end-expiratory pressure, 5 cmH_2_O 19. Anaesthesia was maintained with 1.5 volume% of the inhalational anaesthetic agent isoflurane (Hana Pharm Co. Ltd., Seoul, Korea) via the catheter.

The expression of exosomes, before isoflurane anaesthesia from Control group and after isoflurane anaesthesia from Anaesthesia group, respectively, was determined in the bronchoalveolar lavage (BAL) fluid, as well as blood and tissues from the lung and the brain.

### Groupings

The rats were randomly allocated into two groups, Control group and Anaestheis group, respectively, and were connected by the intubation catheter to the ventilator; those in the Control group received no inhalational anaesthetic agent, whereas those in the Anaesthesia group were treated with 1.5 volume% isoflurane for 2 hours.

### Preparation of BAL, blood, and tissues

The samples were collected as below order: BAL, blood, lung and brain.

After confirming the correct position of the intubation catheter in Control group and after anaesthetic maintenance with 1.5 volume% isoflurane for 2 hours in Anaesthesia group, respectively, cold phosphate-buffered saline (PBS) 1 ml was administered into the catheter. BAL fluid was obtained with the syringe after waiting 3 seconds. The process was repeated eight times. The fluid from BAL was stored at -80°C. The thoracic cavity was exposed and cardiac puncture was performed to collect the blood. The collected blood was transferred to two 10-ml ethylene-diamine-tetraacetic acid (EDTA) tubes (BD Vacutainer) to analyse the exosomes and cytokines, separately. After collection of the blood, 1× PBS 100 ml was injected via the left ventricle and 4% paraformaldehyde 200 ml was injected until whole blood flowed from the right atrium. The lungs and the brain were obtained, and fixed in 4% paraformaldehyde. The fixed tissues were used to detect exosomes by immunohistochemistry. The tissues were dehydrated, paraffinized and embedded in paraffin blocks. The tissues were mounted on 5-μm thick saline-coated slides, using a microtome.

### Exosomes in BAL

The stored BAL fluid was thawed, transferred to a conical 15-ml tube and centrifuged for 10 minutes at 4°C. The supernatant was transferred to a new 1.5-ml Eppendorf tube (Corning Inc., Corning, NY, USA) and centrifuged for 10 minutes at 4°C. The supernatant was filtered through a 0.2-µm sterile syringe filter. The supernatant was kept on ice. Centrifugal filter units (Amicon^®^ Ultra-15 Centrifugal Filer Units; Sigma-Aldrich, St. Louis, MO, USA) with dimension with length, 12.1 cm, diameter, 2.97 cm, filtration area 7.6 cm^2^, minimum final concentration volume, 200 µl and volume 15 ml were equilibrated with sterile PBS and centrifuged for 10 minutes at 4°C. The PBS was discarded after equilibration and centrifugation. The supernatant was filled in the devices and centrifuged at 3000 × g for 30 minutes at 4°C. The retentate was washed with sterile PBS by repetitive pipetting and centrifuged for 30 minutes at 4°C. The concentrated exosomes were collected with a pipette 18,20. The exosomes were analysed with a bead-based flow cytometry assay 21. They were incubated with aldehyde/sulphate-latex beads (Invitrogen, Carlsbad, CA, USA) for 15 minutes at room temperature. After incubation, they were suspended in 1 ml bead-coupling buffer (BCB; PBS + 0.1% bovine serum albumin + 0.01% sodium azide). The samples were incubated overnight at room temperature on a rotating table. The exosome-coated beads were centrifuged for 10 minutes at 4°C, and the supernatant was discarded. The beads were stained with the exosome markers, CD63 (Antibodies-online, Seoul, Korea) and CD81 (Antibodies-online) or rabbit immunoglobulin (Ig)G isotype control (Antibodies-online) for 30 minutes at 4°C. They were washed with BCB and centrifuged. The beads were incubated with AlexaFluor 488 conjugated goat anti-rabbit IgG (H+L) secondary antibody (ThermoFisher Scientific, Waltham, MA, USA) for 30 minutes. The beads were washed with BCB and centrifuged. Finally, the beads were analysed with the BD Accuri C6 flow cytometer (BD Biosciences).

### Exosomes in the blood 22-24

The blood collected by cardiac puncture was used within 30 minutes to prevent degradation and loss of exosomes. Blood samples in EDTA tubes were centrifuged at 1200 × g for 15 minutes at room temperature. The upper plasma layer was transferred to a 15-ml conical tube and centrifuged at 1500 × g for 15 minutes at room temperature to remove the remaining platelets and cell debris. The supernatant was transferred to a 1.5-ml Eppendorf tube, and the exosomes were isolated directly by size exclusion chromatography with Sepharose^®^ CL-2B (Sigma-Aldrich, St. Louis, MO, USA) 21,25. CL-2B was stacked to 10 ml in a 20-ml syringe, and PBS was loaded slowly to wash the CL-2B. The supernatant (plasma) in the Eppendorf tube was gently loaded on top of the washed CL-2B in the 20-ml syringe. The eluted fluid was collected in an Eppendorf tube at the bottom of the 20-ml syringe with no filtration. The fluid was analysed by bead-based flow cytometry, using the same technique as for exosomes in BAL.

### Exosomes in the lung and brain 26,27

The slides for the lung and the brain were deparaffinized with xylene and dehydrated through a gradient (100%, 90%, 80%, and 70%) ethanol series. The slides were immersed in 3% H_2_O_2_ (Sigma-Aldrich, St. Louis, MO, USA) for 10 minutes at room temperature to inhibit endogenous peroxidase activity and incubated with 10% normal goat serum for 1 hour at room temperature. The slides were washed three times in PBS for 5 minutes and incubated with CD63 and CD81 antibodies in 1× PBS with 5% normal goat serum for 1 hour at room temperature. After the incubation, the slides were washed three times with PBS for 5 minutes and incubated with biotinylated secondary antibody, goat anti-rabbit IgG (H+L) (Vector Laboratories, Burlingame, CA, USA) for 1 hour at room temperature. The slides were washed three times with PBS for 5 minutes and incubated with avidin-biotin-peroxidase complex solution (Vector Laboratories) for 1 hour at room temperature. The slides were washed three times with PBS for 5 minutes and treated with DAB solution [DAB (3, 3-diaminobenzidine) peroxidase (HRP) substrate kit (with nickel)] (Vector Laboratories) for 2 minutes. The slides were immersed in 70%, 80%, 90%, and 100% ethanol, as well as xylene, successively. The expression of the exosome markers CD63 and CD81 on the lung and the brain was detected by ImageJ software.

### Cytokines in the blood

Interleukin-2, interferon-γ, tumour necrosis factor (TNF)-α and transforming growth factor (TGF)-β in the blood were assessed by enzyme-linked immunosorbent assays 17. The plasma collected form the blood was transferred into 96-well plate and incubated for 2.5 hours at room temperature. After incubation, the samples were discarder and all wells were washed four times, using 1× wash solution. After washing, biotinylated detection antibody was added to each well and incubated for 1 hour. After incubation, the wells washed with wash buffer. After four times washing, horseradish peroxidase (HRP) solution was added, incubated for 45 minutes and washed with wash solution. After washing, 3,3′,5,5′-tetramethylbenzidine (TMB) solution was added, incubated for 30 minutes in the dark and stop solution was added. The expression of cytokine was read, using microplate reader, at 450 nm.

### Statistical analyses

The primary outcome was the expression for exosome markers in the blood. Blood CD63 and CD81 expression were confirmed in a pilot study of six rats. In the pilot study, the expression levels of CD63 in Control group (n = 3 rats) and Anaesthesia group (n = 3 rats) were 0.078 ± 0.057 and 0.180 ± 0.036, respectively; the CD81 expression levels in Control group (n = 3) and Anaesthesia group (n = 3) were 0.028 ± 0.034 and 0.245 ± 0.054, respectively. The calculated sample sizes, using power analysis, for the primary outcomes were six for CD63 and three for CD81, respectively, with an α of 0.05 and power of 0.9.

Inter-group differences were analysed with the unpaired *t*-test using GraphPad Prism 7.00 software (GraphPad Software, La Jolla, CA, USA). A *p*-value < 0.05 was considered significant. Data are expressed as mean ± standard deviation (median, interquartile range).

### Transmission Electron Microscopy (TEM) for the confirmation of the exosomes

For confirmation of the exosomes in the blood, TEM was performed. The blood sample from cardiac puncture was stored in 1.5-ml Eppendorf tube and transported at 4°C in the ice pack contained box to Institute of Molecular Biology & Genetics in Seoul National University for TEM, using JEM-2100 (JEOL Ltd. Japan) with 200 kV analytical electron microscope.

## Results

Twelve rats were evenly allocated into the two groups without any dropout.

**Figure 1:**
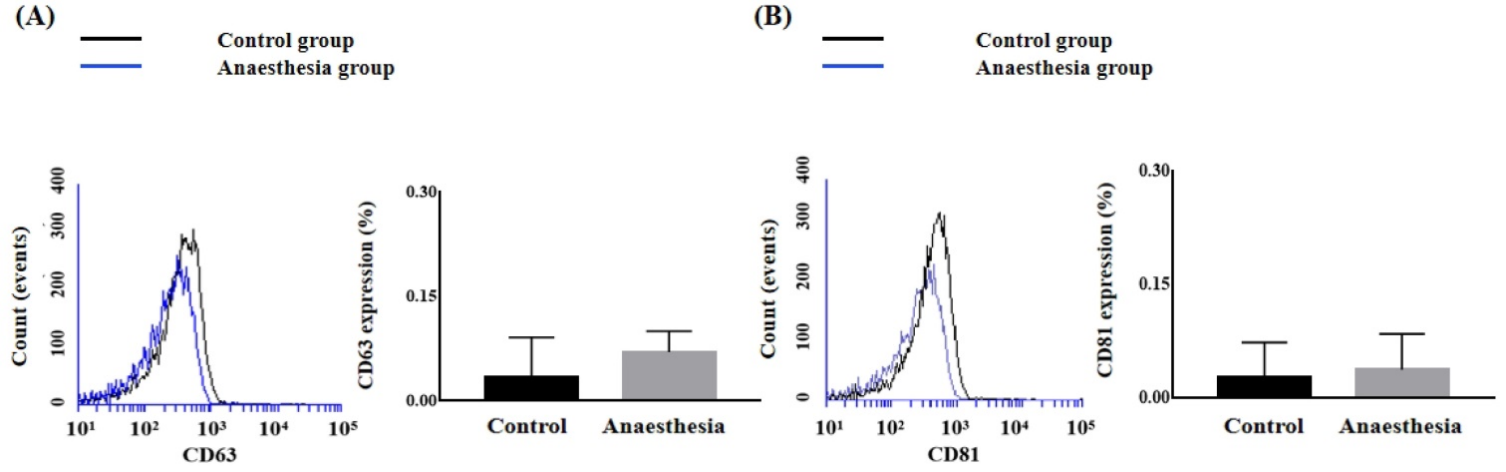
** The expression of exosomes in bronchoalveolar lavage.** (**A**) Cluster of differentiation (CD) 63 (*p* = 0.38), (**B**) CD81 (*p* = 0.81).

The expression levels of the exosome markers CD63 and CD81 in BAL fluid did not differ before and after anaesthesia (CD63, 0.033 ± 0.058% in Control group *vs.* 0.070 ± 0.030% in Anaesthesia group, *p* = 0.38; CD81, 0.027 ± 0.046% in Control group *vs.* 0.037 ± 0.047% in Anaesthesia group, *p* = 0.806) (Figure 1).

**Figure 2:**
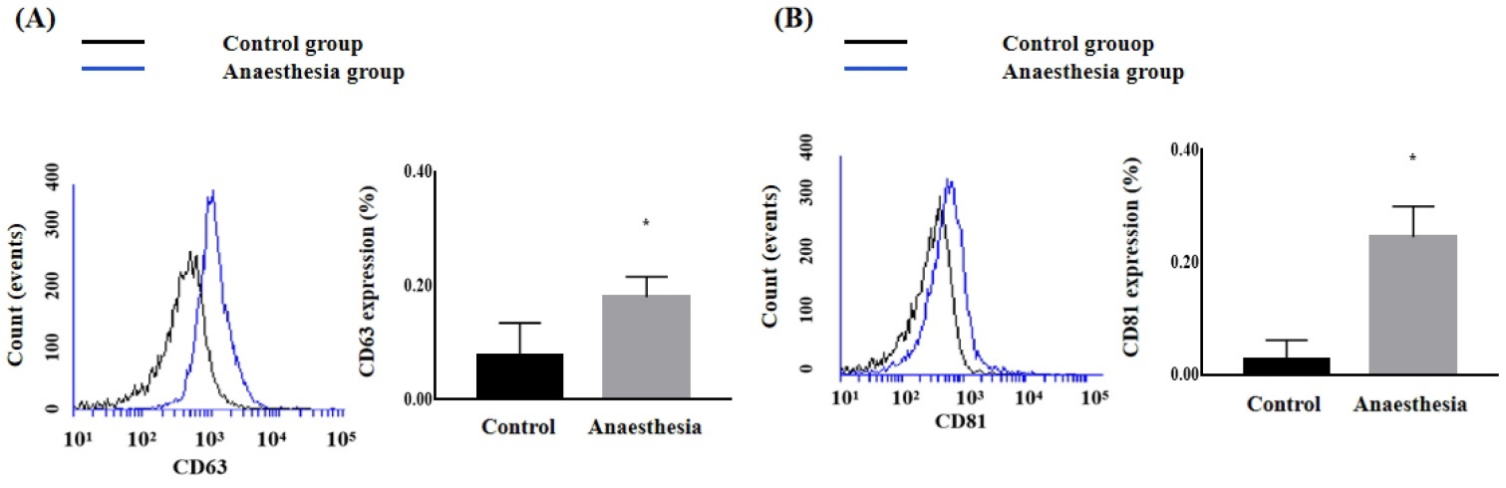
** The expression of exosomes in blood.** (**A**) Cluster of differentiation (CD) 63, (**B**) CD81. ^*^p < 0.05 compared with Control group.

The CD63 and CD81 expression levels in the blood were significantly higher after anaesthesia than before anaesthesia (CD63, 0.078 ± 0.057% in Control group *vs.* 0.180 ± 0.036% in Anaesthesia group, *p* = 0.02; CD81, 0.028 ± 0.034% in Control group *vs.* 0.245 ± 0.054% in Anaesthesia group, *p* < 0.01) (Figure 2).

**Figure 3:**
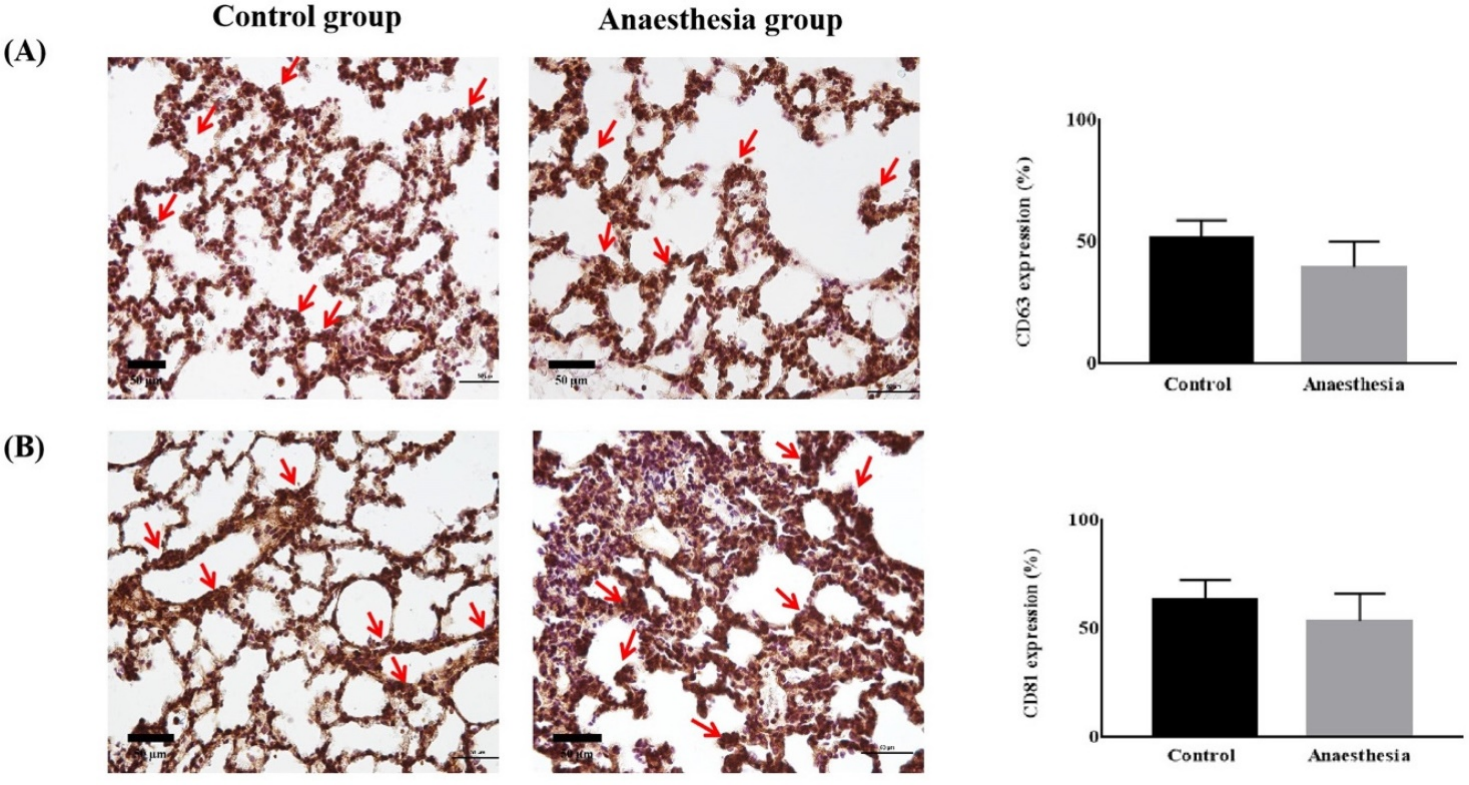
** The expression of exosomes in lung tissue.** (**A**) Cluster of differentiation (CD) 63 (*p* = 0.12), (**B**) CD81 (*p* = 0.24). The arrows indicate CD63 or CD81.

CD63 and CD81 expression did not differ in lung tissues before and after anaesthesia (CD63, 51.39 ± 7.46% in Control group *vs.* 39.60 ± 10.56% in Anaesthesia group,* p* = 0.118; CD81, 63.52 ± 8.94% in Control group *vs.* 53.43 ± 12.77% in Anaesthesia group, *p* = 0.243) (Figure 3).

**Figure 4:**
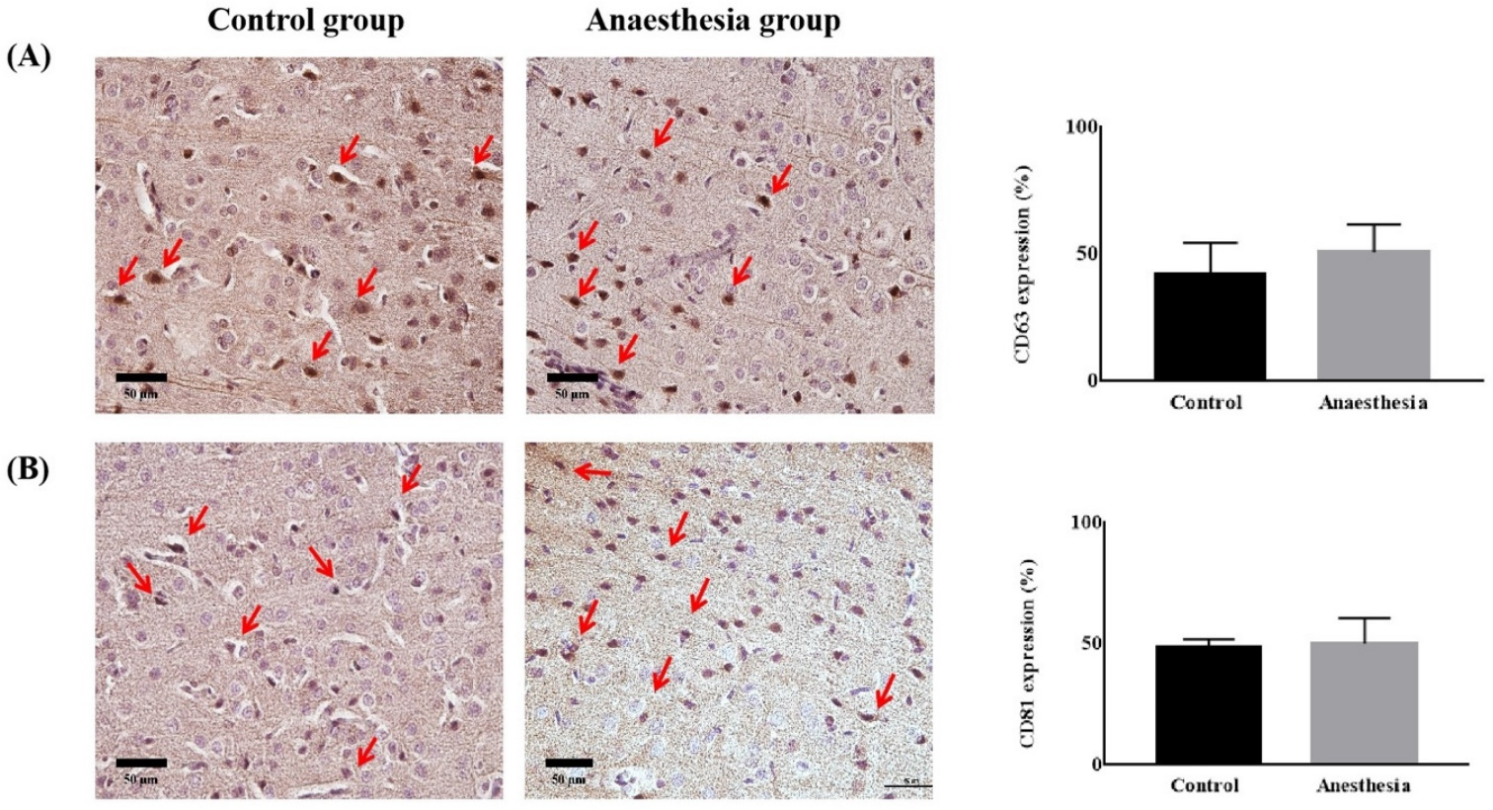
** The expression of exosomes in brain tissue.** (**A**) Cluster of differentiation (CD) 63 (*p* = 0.32), (**B**) CD81 (*p* = 0.74). The arrows indicate CD63 or CD81.

CD63 and CD81 expression in the brain did not differ before and after anaesthesia (CD63, 41.84 ± 12.67% in Control group *vs.* 50.81 ± 10.79% in Anaesthesia group, *p* = 0.322, CD81, 48.27 ± 3.62 % in Control group vs. 50.18 ± 10.41% in Anaesthesia group, *p* = 0.740) (Figure 4).

The cytokines in the blood did not have any significant difference between the two groups (Table 1).

**Figure 5:**
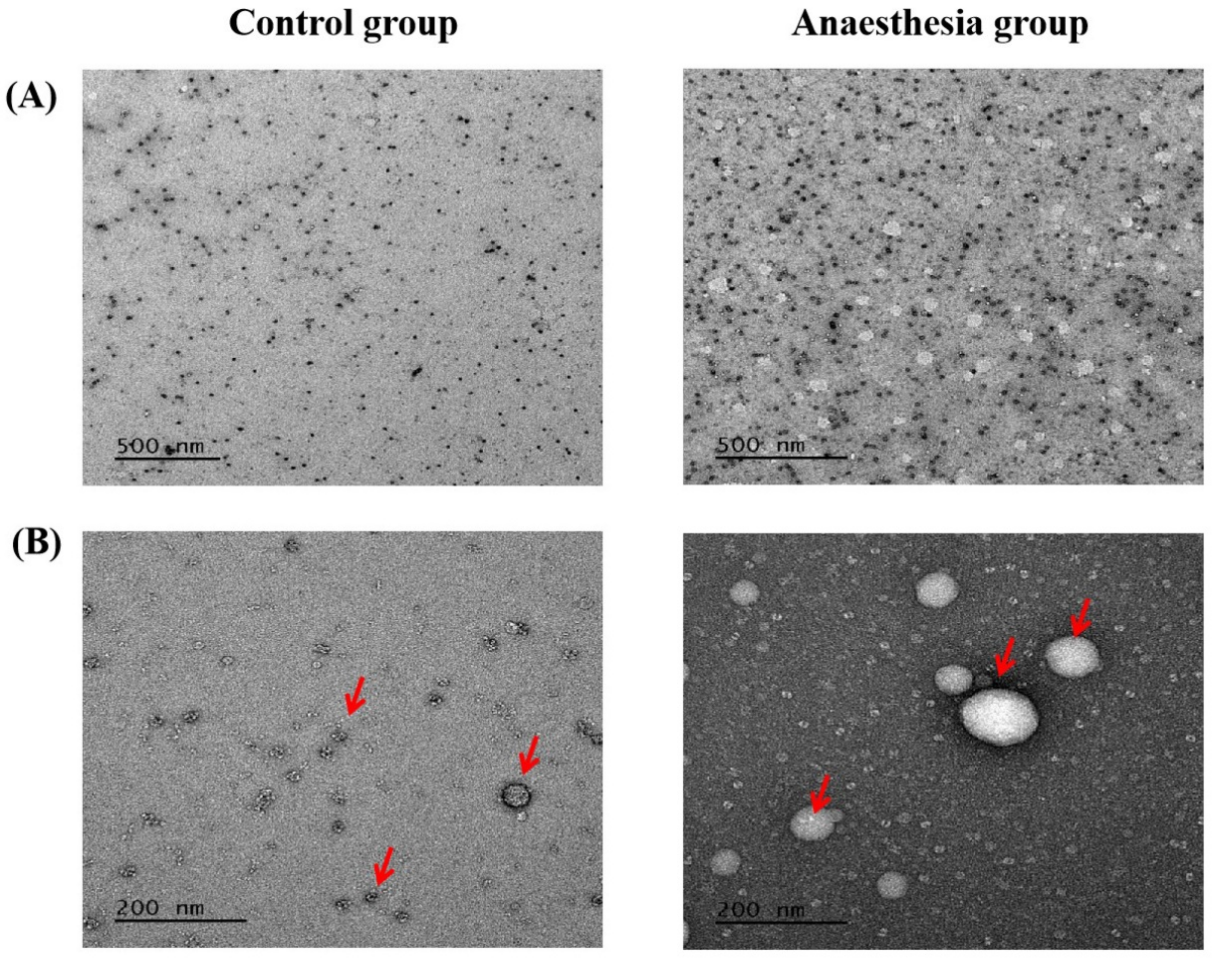
** Exosomes in electron microscopy.** (**A**) Scale bar, 500 nm, (**B**) Scale bar, 200 nm. The arrows indicate exosomes.

Electron microscopy confirmed the exosomes (Figure 5).

## Discussion

The present study demonstrated that the expression levels of CD63 and CD81 as markers for exosomes in the blood increased after isoflurane anaesthesia. However, their increased expression was not detected in the BAL fluid, the lung, or brain tissues.

The mechanism of inhalational anaesthetic agents remains controversial. It is known to be associated with neurotransmitters and their related ion channels, which induce anaesthetic effects, including hypnosis on target organs 28. Therefore, numerous studies have researched the effect of inhalational anaesthetic agents on specific neurotransmitter-related ion channels for target organs, including ligand-gated ion channels, such as gamma-aminobutyric acid receptors in the brain and voltage-gated calcium channels in the heart 29,30. However, the studies have been limited to individual target organs and do not explain the effect on the entire body. They have investigated the effects of an inhalational anaesthetic agent on neurotransmitter-related ion channels on individual target organs. In contrast, the present study investigated increased CD63 and CD81 expression levels in the blood after isoflurane anaesthesia; these agents might activate specific neurotransmitters and their related ion channels in the whole body, although more research is needed for confirmation. Therefore, exosomes may more broadly affect the entire body, not a specific target organ. When exosomes were first discovered, they were thought to be “garbage disposals” that functioned by eliminating unwanted cellular components. However, the cell-specific cargo of proteins, lipids, and genetic material in exosomes are transported as a Trojan horse to recipient cells where they induce biological processes 31 depending on their origins. Therefore, exosomes affect specific target organs 32, regardless of the existence of neurotransmitter-related ion channels on the target organ.

Cells continuously maintain homeostasis. Anaesthesia itself produces stress, which can interrupt homeostasis, although anaesthesia is unavoidable for some diagnostic or therapeutic procedures 33. The increased CD63 and CD81 expression levels in the blood after isoflurane anaesthesia in the present study may be a result of interrupted homeostasis. Numerous reports on changes in homeostasis in patients with various diseases after anaesthesia have been published 34,35. However, they have failed to clarify the definite causes of the clinical implications from anaesthesia, except anaesthesia itself, although they have shown specific clinical changes to target organs after general anaesthesia. The increase in the CD63 and CD81 expression levels without any change in cytokines after isoflurane anaesthesia in the present study may help clarify the underlying mechanism. Similar results were observed in our previous study; endoplasmic reticulum stress increased after isoflurane anaesthesia, although no changes in blood cytokine levels were observed 17. We also observed no differences in immunohistochemistry of the brain, liver, or kidneys before and after isoflurane anaesthesia 17. We expected changes in all samples including the BAL fluid, blood, and brain. However, we only detected changes in the blood, indicating that blood is more sensitive to the effect of isoflurane anaesthesia than the lungs and brain.

Exosomes are surrounded by surface markers called tetraspanins. CD9, CD63, and CD81 are the classical surface markers for exosomes. Several studies have confirmed that they are exosome markers 21, 36-38 and that they are dependent on size. CD9 is expressed in vesicles < 30 nm, although its expression is controversial if vesicles < 30 nm, and CD63 and CD81 are mainly expressed in vesicles >100 nm 39. Detecting CD9 requires a specific technique because of its size, such as one-step density gradient ultracentrifugation 39. Therefore, we did not confirm CD9. A specific technique to detect CD9 would provide more concrete information on the effect of isoflurane anaesthesia on the expression of exosomes.

Isolation of exosome, using surface marker, was controversial but has been commonly used. In the present study, we performed electron microscopy to confirm the expression and the size of exosome.

To get the pure exosomes from the immune cells, exosomes from the platelets should be deleted, using CD61 MicroBeads. Unfortunately, we did not perform the procedure. However, the portion of exosomes from the platelet was known to be limited. Therefore, the impact on the expression of exosome from platelet in the present study might be limited.

The most popular method to isolate exosomes is ultracentrifugation with or without modifications 37. However, the main disadvantage is low yield due to damage to the exosomes. Therefore, we used size exclusion chromatography in the present study.

As mentioned above, the effects of the inhalational anaesthetic agent itself on exosomes should be verified without influence from a specific target organ. For example, inhalational anaesthetic agents have hypnotic and hemodynamic effects, which may be associated with the results of the present study. Therefore, the results should be compared with the same hemodynamic changes without isoflurane to confirm the pure effect of an inhalational anaesthetic agent.

On the contrary with the results in the present study, recently, Bleilevens et al. recently reported that repetitive treatment of inhalational anesthetics did not have any impact on the expression of exosomes 40. They administered isoflurane at three time per week for 4 weeks or 8 weeks to get anaesthetic-induced preconditioning against ischemic injury. They maintained anaesthesia with isoflurane 1.3 volume% but they used anaesthesia chamber, not intubation catheter, to administer isoflurane. It meant that there was the possibility that less concentration of isoflurane might be administered. Moreover, Bleilevens et al. maintained anaesthesia just for 30 minutes, although they performed anaesthesia at three time per week for 4 weeks or 8 weeks. These factors might be associated with the different results from the results of the present study.

The interpretation of the results with the limited expression of exosomes only in the blood should be considered with the use of the mixture, the surgical incision and the time to sacrifice the experiments in the study. We intraperitoneally administered a mixture of ketamine and xylazine to induce anaesthesia in both groups. Therefore, all experiments were affected by the intraperitoneal administration of the ketamine and xylazine mixture. We wanted to build study design to evaluate the pure effect of an inhalational anaesthetic agent on the expression of exosomes without any ethical problem. For the purpose, any surgical incision, except the procedure for prevention of air leakage from airway, was not allowed. The procedure was inevitable to guarantee the confirmation of delivery of isoflurane to respiratory system. Although the procedure could lead to the change of the expression of exosomes, it was performed in the both groups and the impact on the expression of exosome would be same in the both groups. If we induced anaesthesia with a short-acting inhalational anaesthetic agent, using anaesthesia induction chamber, instead of the mixture of ketamine and xylazine, several problems were remained, although a short-acting inhalational anaesthetic agent was isoflurane. Firstly, we could not guarantee the similar concentration of a short-acting inhalational anaesthetic agent between the two groups because tidal volume of individual was different, according to the rat, although the weight was similar. Secondly, the impact of a short-acting inhalational anaesthetic agent on respiratory system during anaesthesia induction with ventilation should be considered. Thirdly, we could not rule out the effect of the short-acting inhalational anaesthetic agent. If we performed intubation without any anaesthetic agent to induce anaesthesia, it had an ethical problem and anatomical damage on respiratory system. For the exact effect of an inhalational anaesthetic agent on the expression of exosomes, the time to sacrifice should be same. We maintained anaesthesia with isoflurane for 2 hours to get the enough time to impact on the body and rule out the effect of the mixture for anaesthesia induction. If Control group was sacrificed at the same time, it also had an ethical problem because Control group had no anaesthesia maintenance. Moreover, inappropriate anaesthetic depth and early emergence from anaesthesia resulted in the damage on respiratory system. Therefore, we used the mixture to induce anaesthesia and inevitably sacrificed the rats at different times. However, the effect of the mixture of ketamine and xylazine for anaesthesia induction is usually maintained for 30 minutes 41,42, and the time from the administration of the mixture to to the confirmation of intubation and the procuration of the samples took at least 20 minutes and within 30 minutes in the study. Therefore, the effect of the mixture on the expression of exosomes might be limited.

Although the present study did not give the information for the expression of exosomes in general anaesthesia with or without isoflurane, it showed the changes for the expression of exosomes before and after general anaesthesia with isoflurane.

In conclusion, general anaesthesia with an inhalational anaesthetic agent increased the expression of exosomes in the blood. However, the change was limited to the blood and was not observed in the alveoli or the brain. These findings might be clues to explain the effects of inhalational anaesthetic agents on the whole body, instead of neurotransmitters and their related ion channels on a specific individual target organ.

## Figures and Tables

**Table 1:** Cytokines in the blood

	Control group	Anaesthesia group	*p-*value
IL-2 (ng/mL)	103.10 ± 34.68 (105.90, 69.23-134.20)	95.45 ± 38.30 (100.70, 57.03-128.70)	0.777
IFN-γ (ng/mL)	200.70 ± 13.84 (202.10, 186.70-213.30)	205.30 ± 31.19 (215.50, 172.70-227.80)	0.795
TNF-α (ng/mL)	302.40 ± 48.25 (297.40, 258.70-351.00)	308.80 ± 66.20 (299.60, 250.20-376.50)	0.881
TGF-β (ng/mL)	243.30 ± 14.58 (238.70, 233.00-258.10)	250.30 ± 25.61 (250.10, 225.50-275.10)	0.653

Data is presented as mean ± standard deviation (median, interquartile range). **Abbreviations:** IL, interleukin, IFN, interferon; TNF, tumor necrosis factor; TGF, transforming growth factor.
